# Notes and News

**Published:** 1899-11-04

**Authors:** 


### Notes and News.

(Collected and Communicated.)

A meeting was held at the Memorial Hall last week to consider the suggested garden city scheme for relieving the congested centres of population. The idea is to establish half-a-dozen or more garden cities, not as mere suburbs of London, but at least 40 or 50 miles from it, with the object of removing manufacturers and their people to better and more roomy sites.

The foundation-stone of the new St. Mary's Hospital at Manchester has been laid by the Countess of Derby. The institution will be situated in Gloucester and Oxford Streets, and it will accommodate 125 patients. The largest portion of the space will be reserved for women, and provision will be made for the training of medical students, nurses, midwives, and monthly nurses. The cost of the building will be about £54,000, whilst £16,000 has been paid for the site. A deficiency of £11,000 remains to be provided.

At last the Manchester Corporation has received the permission of the Local Government Board to raise a loan for the purpose of constructing filter beds for the bacterial treatment of the sewage of that city. Certain conditions are attached, among which are that an area of not less than 92 acres of filter beds be provided for the treatment of sewage by double contact; that these filter beds be worked in cycles of not less than eight hours; that provision be made for the land treatment of storm-water sewage up to six times the volume of the dry weather flow, and that so far as levels permit the effluent from the bacteria beds shall flow on to the land.

In relation to this matter it is worth noting that the scheme for the bacterial treatment of the sewage of Hampton, which has been working satisfactorily for the past ten months, has recently received the public sanction of the Thames Conservancy Board, their chairman (Sir F. Dixon-Hartland, M.P.) having just formally opened the works. In this case the sewage is raised by air pressure on the Shone hydro-pneumatic system, and is then treated on bacteria-beds arranged in groups of three, on each of which it lies for the space of about an hour. Each bed is worked three times in the 24 hours; the same time as is provided for in the conditions applying to the Manchester scheme—namely, in cycles of eight hours. The effluent from the filter beds is used for irrigation purposes.

It is satisfactory to note that the conduct of the Boers under General Joubert towards the wounded who have fallen into their hands seems to have been characterised by consideration.

We regret to learn that Mr. George Scudamore, who for 27 years has been the active and competent secretary of the Samaritan Free Hospital, and also for the past 33 years of the St. Marylebone Female Protection Society, has been compelled to resign his position as secretary to both institutions on account of ill-health. It was owing to his untiring efforts that the present Samaritan Hospital was built.

A report of the Water Committee of the London County Council urges that the analyses of samples of water drawn from the companies' mains once a month or even once a week, as now performed on behalf of the Local Government Board, are not enough, and that the only way to ensure a high state of purity would be by a daily examination of the water as delivered to the consumer.

In the annual report of the Local Government Board, which has just been issued, attention is drawn to the fact that adulteration of milk by the addition of water still remains a very common practice. The inspectors under the Sale of Food and Drugs Act have done something, but it can hardly be considered a satisfactory state of affairs when we find that one in every eight of the samples of milk analysed in London is reported as showing evidence of dilution. It is pointed out, however, that children brought up even on this diluted milk fare better than those fed with the condensed separated milk which is now very commonly sold. This, we are told, contains so little nutriment that the contents of ten half-pound tins would have to be consumed in order to obtain the equivalent of a pint of good milk. Of course, this means when its food value is estimated according to the quantity of fatty matter contained in it. It is satisfactory to learn that several local authorities have issued warnings in regard to the danger of half-starving infants by feeding them with this stuff.

Mr. Andrew Clark has written a strong letter to the *Times*, urging the claims of medical witnesses in regard to remuneration in criminal cases. He points to the substantial grievance which is done to medical men, who are compelled to leave their practices, sometimes for a week at a time, and stay at an assize town for the purpose of giving evidence on behalf of the Crown. The same injustice, he says, is not done either in Scotland or in Ireland, the arrangement in Scotland being that a doctor called by the Crown receives £2 2s. a day, with one day added on, so that he gets £4 4s. for one day, £6 6s. for two days, and so on. In Ireland, when a medical witness is summoned to attend at sessions in the town in which he resides, the usual fee of £1 1s. is paid, and if he be detained more than three hours an additional fee of £1 1s. is paid. When summoned to any other town £2 2s. a day and travelling and hotel expenses are allowed. What the Joint Committee of the British Medical Association and the United Kingdom Police Surgeons' Association ask is £2 2s. a day, with first-class railway fare.

